# Optimal targets of chronic kidney disease-mineral and bone disorder markers for Chinese patients with maintenance peritoneal dialysis: a single-center retrospective cohort study

**DOI:** 10.1080/0886022X.2022.2041438

**Published:** 2022-04-05

**Authors:** Limeng Chen, Xueqing Tang, Hua Zheng, Haiyun Wang, Peng Xia, Ying Wang, Xue Zhao, Zijuan Zhou, Ling Qiu, Xuemei Li

**Affiliations:** a Department of Nephrology, Peking Union Medical College Hospital, Chinese Academy of Medical Sciences, Beijing, China; b Department of Internal Medicine, Peking Union Medical College Hospital, Chinese Academy of Medical Sciences, Beijing, China; c Clinical laboratory, Peking Union Medical College Hospital, Chinese Academy of Medical Sciences, Beijing, China

**Keywords:** CKD-MBD, PTH, peritoneal dialysis, survival analysis, hyperparathyroidism

## Abstract

**Background:**

The chronic kidney disease-mineral and bone disorder（CKD-MBD) is known to be associated with increased mortality in dialysis patients, but whether current global guidelines for CKD-MBD, which were primarily developed from hemodialysis, are suitable for peritoneal dialysis (PD) patients practice require further investigation.

**Methods:**

This is a single-center retrospective cohort study. In total 491 prevalent PD patients (median follow-ups: 34 months) from Peking Union Medical College Hospital (PUMCH) from January 2004 to December 2017 were included and followed until 30 June 2018. In the first dialysis year, the average levels of serum calcium, albumin-corrected calcium (CorCa), phosphorus, and parathyroid hormone (PTH) levels were the interested predictors in Cox proportional regression model.

**Results:**

Of these PD patients (age 58 ± 17 years), 52% were male and 36% had diabetic nephropathy. In Cox regression over first-year mean parameters, PTH <100 pg/mL (HR = 1.97, 95% CI 1.32 to 2.94, *p* < 0.001) and ≥300 pg/mL (HR = 2.24, 95% CI 1.32 to 3.81, *p* = 0.003) were associated with increased all-cause mortality than that of PTH 100–200 pg/mL. Patients with albumin-corrected serum calcium level < 2.13 mmol/L also had higher risk of death than patients with level of 2.13 to 2.38 mmol/L (HR = 2.06, 95% CI 1.06 to 4.01, *p* = 0.02). Serum phosphorus ≥1.45 mmol/L were associated with increased all-cause mortality. However, lacking of data on 25-hydroxy vitamin D, alkaline phosphatase, and activated vitamin-D are limitations of our analysis.

**Conclusions:**

As one of the largest PD cohort study focusing on CKD-MBD, we demonstrated that the level of CKD-MBD markers in the first PD year are independent predictors of all-cause mortality. PTH 100–300 pg/mL might be the best target for Chinese PD patients.

## Introduction

Chronic kidney disease-mineral and bone disorder (CKD-MBD) is one of the most common complications in patients with CKD, especially in dialysis patients. The serum makers of CKD-MBD, namely, serum calcium, phosphorus and parathyroid hormone (PTH), have been well proved to be associated with cardiovascular outcomes and mortality in dialysis patients [1–5]. Although target ranges for CKD-MBD markers have been proposed in several international guidelines [6,7], these were mostly based on data from hemodialysis (HD) patients and few Chinese patients were involved.

There are a few studies about CKD-MBD in peritoneal dialysis (PD) patients [8,9]. One study from the USA illustrated a *U*-shaped association between PTH concentration and mortality in PD patients. PTH concentrations of less than 200 pg/mL and more than 700 pg/mL were associated with increased mortality, which was not observed in HD patients [10]. Recently, data from Taiwan demonstrated that serum calcium level of ≥2.38 mmol/L (9.5 mg/dL) and serum phosphorus levels of either ≥1.63 mmol/L (6.5 mg/dL) or <1.13 mmol/L(3.5 mg/dL) were associated with increased mortality. But PTH levels ≥600 pg/mL were associated with lower mortality compared to reference range (150–600 pg/mL), while PTH levels lower than reference range (<150 pg/mL) showed insignificantly increased mortality [11]. This result differed from the reports that had contributed to KDIGO and JSDT (Japanese Society of Dialysis and Transplantation) and also inconsistent from a study in Taiwan HD patient [12]. Since target ranges of CKD-MBD markers varied among regions, races and dialysis modalities, recommendation focusing on Chinese PD population would be helpful in this specific population. To that end, we analyzed data from our established PD cohort to explore the role of CKD-MBD markers including calcium, phosphorus, and PTH on all-cause mortality in maintenance PD patients, providing evidence for establishing optimal targets of CKD-MBD markers for Chinese PD patients.

## Materials and Methods

### Study Design and Participants

**Figure 1.**
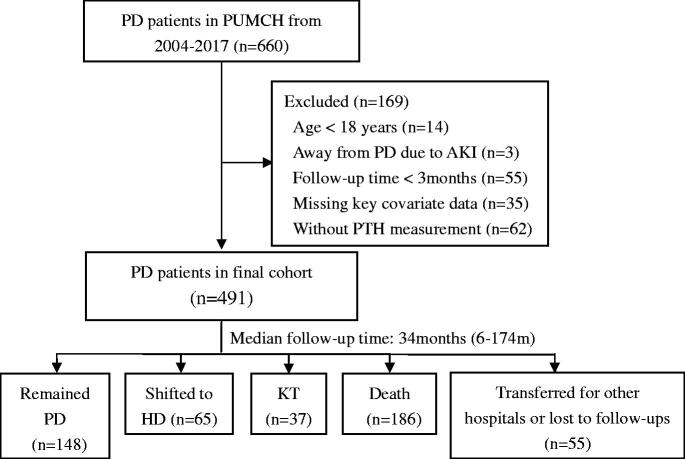
Study Cohort Creation. PD: peritoneal dialysis; PUMCH: Peking Union Medical College Hospital; PTH: parathyroid hormone; HD: hemodialysis; KT: kidney transplant.

This was a single-center, retrospective cohort study. We investigated patients in the PD center of Peking Union Medical College Hospital (PUMCH), who were registered from 1 January 2004 to 31 December 2017. Patients were excluded if they were less than 18 years old, or quitted PD due to renal recovery, or had less than 3months follow-up time, or had missed key covariate data and CKD-MBD marker measurement (Figure 1). This study was approved by PUMCH ethical review committee [ZS-1152]. Given the nonintrusive nature of the research, the requirement for written consent was waived by the Review Board.

Finally, 491 prevalent patients were enrolled in the current study. Among these patients, 148 patients remained on PD, while 65 patients shifted to HD and 37 patients received kidney transplantation by the end of June 2018. Meanwhile, 186 patients died and 55 patients transferred to other hospitals during the 34 months (6 to 174 months) of median follow-up time.

### Clinical Characteristics

Demographics and baseline clinical laboratory data were recorded, including age, gender, primary kidney disease, comorbidity including diabetes and hypertension, residual glomerular filtration rate (GFR) [13], peritonitis, duration of PD, body mass index (BMI), hemoglobin (Hb), low density lipoprotein (LDL), serum total calcium (Ca), serum phosphorus (P), parathyroid hormone (PTH), peritoneal equilibration test (PET), urea clearance index (Kt/V), creatinine clearance (CCr) and residual renal glomerular filtration rate (rGFR). Total serum calcium was corrected using the following formula: corrected calcium (CorCa)=(0.02 × [normal albumin level − exact albumin level])+measured serum calcium. The baseline values of Ca/CorCa, P and PTH were defined as the average value in the first PD year.

### Laboratory Methods

Complete blood counts, electrolytes, albumin, renal function, and prescription were measured and reviewed monthly. Other biochemical indexes and dialysis adequacy were assessed every 3 months at least. Blood samples were drawn using standardized techniques and were transported to the laboratory immediately, where they were measured using automated and standardized methods. The serum intact PTH was measured using “Third-generation immunoradiometric intact PTH assay” (IMM2000, SIEMENS, Germany).

### Definition of Survival Outcomes

The main outcome was all-cause mortality, predominantly consisting of infection and cardiovascular death (myocardial infarction, congestive heart failure, arrhythmia, stroke, and peripheral arterial disease). In survival analysis, we censored follow-up at shifting to HD treatment, kidney transplantation, loss to follow-up, or the end of the study (30 June 2018).

### Statistical Analysis

Descriptive statistics are expressed as frequencies (percentages) for categorical variables, and as mean ± standard deviation for continuous variables, except for rGFR, the incidence of peritonitis, PD vintage, and PTH level, which were given as a median and interquartile range because they were highly skewed. *T-* tests, Mann–Whitney *U* tests, or chi-square tests were used as appropriate for statistical significance testing.

Cox proportional regression model and Kaplan–Meier method were performed with all-cause mortality. For the Kaplan–Meier analysis, the significance of survival differences was evaluated using the log-rank test. Multiple comparisons of Kaplan-Meier curves were conducted with Sidak correction. To build the multivariate Cox regression model, all potential predictors variables were evaluated for association with mortality using the univariate Cox regression method. Serum concentrations of calcium/CorCa, phosphorus, and PTH were considered as potential predictors. The pre-defined criteria for inclusion in the multivariate Cox regression model consisted of a univariate p-value <0.05. A multivariate Cox regression model with the potential predictors represented as categorical variables was performed. Considering the U-shaped association between CKD-MBD markers and mortality [10,11] and the reference range provided in international guidelines [6,7], PTH values were categorized into 4 groups (<100, 100–200, 200–300, and ≥300 pg/mL). Similarly, calcium/CorCa values were categorized into 4 groups (<2.13, 2.13–2.38, 2.38–2.63, and ≥2.63 mmol/L; OR <8.5, 8.5–9.5, 9.5–10.5, and ≥10.5 mmol/L respectively) and phosphorus values were also categorized into 3 groups (<1.13, 1.13–1.45, ≥1.45 mmol/L; OR <3.5, 3.5–4.5, and ≥4.5 mg/dL respectively), where 1.45 and 1.13 mmol/L were the higher limit and mid-point of normal serum phosphorus range for Chinese population, respectively.

All statistical analyses were performed using SPSS, version19.0. (SPSS Inc., Chicago, IL, USA).

## Results

### Baseline Demographic, Clinical, and Laboratory Characteristics by PTH level

For 491 included patients with a mean age of 58 ± 17 years, 52% were men, and the median rGFR at the start of PD was 3.8 mL/min/1.73 m^2^. The most common primary disease was diabetic nephropathy (DN) (36%), followed by chronic glomerulonephritis (26%) and hypertension (22%). By the end of Jun 2018, 186 (38%) patients were deceased during a median follow-up of 34 months (6–174 months, interquartile range: 15–55 months) (Figure 1, Table 1).

**Table 1.** Demographic Characteristics and Baseline Laboratory Data of PD Patients Stratified by PTH Level.

	PTH (pg/mL)^a^					
	Total	<100	100–200	200–300	≥300	*p*
Number	491	191	118	91	89	–
Age (year)	58 ± 17	62 ± 15	56 ± 15	53 ± 16	58 ± 16	<0.001
Male (%)	52	51	51	57	52	0.79
Primary diseases (%)						
DN	36	41	38	31	28	0.12
HTN	22	21	23	20	27	0.63
CGN	26	21	26	31	33	0.15
Others	16	17	13	19	12	0.48
rGFR(ml/min/1.73m^2^)	3.8 ± 3.0	3.9 ± 3.1	4.0 ± 3.0	4.4 ± 3.7	3.2 ± 2.1	<0.001
BMI（kg/m2)	22.8 ± 3.6	22.4 ± 3.5	22.8 ± 3.9	23.8 ± 3.7	22.3 ± 3.1	0.03
Albumin (g/L)	35.3 ± 13.5	35.3 ± 21.5	36.4 ± 5.6	36.4 ± 5.2	34.8 ± 5.3	0.81
Hb (g/L)	91.7 ± 17.6	93.9 ± 16.1	94.9 ± 16.6	91.3 ± 20.2	84.7 ± 17.6	<0.001
LDL (mmol/L)	2.86 ± 1.02	2.92 ± 1.06	2.82 ± 1.06	2.80 ± 0.98	2.81 ± 1.03	0.78
Ca (mmol/L)^a^	2.25 ± 0.21	2.33 ± 0.20	2.25 ± 0.16	2.23 ± 0.16	2.12 ± 0.25	<0.001
CorCa (mmol/L)^a^	2.38 ± 0.41	2.47 ± 0.19	2.35 ± 0.15	2.29 ± 0.19	2.22 ± 0.25	<0.001
P (mmol/L)^a^	1.58 ± 0.37	1.51 ± 0.34	1.56 ± 0.32	1.62 ± 0.35	1.69 ± 0.42	0.002
PET (Cr 4h D/P-value)*	0.69 ± 0.13	0.70 ± 0.12	0.69 ± 0.14	0.68 ± 0.1	0.70 ± 0.14	0.58
Kt/V*	2.43 ± 0.68	2.46 ± 0.71	2.42 ± 0.64	2.48 ± 0.71	2.42 ± 0.66	0.90
CCr (L/W/1.73m^2^)*	80.7 ± 30.9	83.2 ± 33.8	81.2 ± 29.2	84.7 ± 33.2	75.4 ± 22.9	0.19

Note: Values for categorical variables given as percentage; for continuous variables, as mean ± standard deviation or median (25th percentile, 75th percentile).

PD: peritoneal dialysis; PTH: parathyroid hormone; DN: diabetic nephropathy; HTN: hypertension; CGN: chronic glomerulonephritis. rGFR: residue glomerular filtration rate: body mass index; Hb: Hemoglobin; LDL: low density lipoprotein; Ca: serum total calcium; CorCa: corrected serum total calcium; P: serum phosphorus; PET: peritoneal equilibration test; Kt/V: urea clearance index; CCr: creatinine clearance.

^a^The value of Ca, CorCa, P, PTH using the average value in the first PD year.

*evaluated after 3 months of PD

Among these patients, higher PTH concentration was associated with lower baseline hemoglobin at the baseline. Meanwhile, patients with higher PTH concentration exhibited lower serum calcium/CorCa level and higher serum phosphorus level. There was no significant difference in gender, incidence of peritonitis, albumin, LDL, and the peritoneal transport function, Kt/V, CCr after initial 3 months of PD. All groups achieved the recommended dialysis adequacy (weekly Kt/*V* > 1.7 per week) (Table 1).

### Risk Factors for All-cause Mortality

Based on the result of univariate Cox proportional regression (Supplementary Table 1), the multivariate Cox regression model showed that increased age, comorbidity of diabetes mellitus, peritonitis, lower albumin levels, PTH (<100, or ≥300 pg/ml), hypocalcemia (CorCa < 2.13 mmol/L) and hyperphosphatemia (≥1.45 mmol/L) were associated with increased all-cause mortality but not cardiovascular mortality (data not shown). In contrast to general expectation, neither rGFR nor weekly Kt/V was significant in these multivariate models (Table 2–4).

**Table 2.** Cox Proportional Hazard Ratio for All-cause Mortality by PTH Level in PD Patients

	Crude			Adjusted		
Variables	HR	95% CI	*p*	HR	95% CI	*p*
Age	1.059	1.047–1.072	<0.001	1.063	1.047–1.078	<0.001
DM	2.210	1.676–2.913	<0.001	1.862	1.350–2.568	<0.001
rGFR (ml/min/1.73 m^2^)						
<2	Ref.	–	–	–	–	–
2–5	1.045	0.737–1.483	0.80	–	–	–
≥5	0.958	0.649–1.414	0.83	–	–	–
peritonitis (times per month)	3.978	1.503–2.6326	0.005	68.24	1.907–2442	0.021
Albumin (per g/dL increment)	0.973	0.95–0.996	0.02	0.958	0.931–0.986	0.004
Kt/V (per 1 increment)	0.937	0.756–1.161	0.55	0.863	0.675–1.103	0.238
PTH (pg/mL)^a^						
<100	1.849	1.265–2.704	0.002	1.968	1.317–2.940	0.001
100–200	Ref.	–	–	Ref.	–	–
200–300	1.083	0.655–1.789	0.76	1.426	0.833–2.443	0.196
≥300	1.084	0.660–1.78	0.75	2.243	1.320–3.810	0.003

Note: adjusted for age, diabetes mellitus, eGFR, peritonitis, albumin, KT/V.

PD: peritoneal dialysis; HR: hazard ratio; CI: confidence interval; DM: diabetes mellitus; rGFR: estimated glomerular filtration rate; PTH: parathyroid hormone; Kt/V: urea clearance index.

^a^Using the average value in the first PD year for PTH.

**Table 3.** Cox Proportional Hazard Ratio for All-cause Mortality by CorCa Level in PD Patients

	Crude			Adjusted		
Variables	HR	95% CI	*p*	HR	95% CI	*p*
Age	1.059	1.047–1.072	<0.001	1.061	1.046-1.075	<0.001
DM	2.210	1.676–2.913	<0.001	1.754	1.269-2.424	0.001
rGFR（ml/min/1.73m^2^)						
<2	Ref.	–	–	–	–	–
2–5	1.427	0.197–10.36	0.73	–	–	–
≥5	2.155	0.301–15.425	0.44	–	–	–
peritonitis (per month/time increment)	3.978	1.503–10.526	0.005	61.01	1.273–2924	<0.001
Albumin (per g/dL increment)	0.973	0.95–0.996	0.02	0.959	0.931–0.988	0.005
Kt/V (per 1 increment)	0.937	0.756–1.161	0.55	0.838	0.653–1.077	0.168
CorCa (mmol/L)^a^						
<2.13	1.165	0.626–2.167	0.63	2.057	1.056–4.006	0.034
2.13–2.38	Ref.	–	–	Ref.	–	–
2.38–2.63	1.433	1.024–2.004	0.04	1.291	0.901–1.850	0.165
≥2.63	1.708	1.123–2.597	0.01	1.104	0.702–1.735	0.668

Note: adjusted for age, diabetes mellitus, eGFR, peritonitis, albumin, KT/V.

PD: peritoneal dialysis; HR: hazard ratio; CI: confidence interval; DM: diabetes mellitus; rGFR: estimated glomerular filtration rate; CorCa: corrected total serum calcium; Kt/V: urea clearance index.

^a^ Using the average value in the first PD year for CorCa.

**Table 4.** Cox Proportional Hazard Ratio for All-cause Mortality by P Level in PD Patients

	Crude			Adjusted		
Variables	HR	95% CI	*p*	HR	95% CI	*p*
Age	1.059	1.047–1.072	<0.001	1.064	1.048–1.079	<0.001
DM	2.210	1.676–2.913	<0.001	1.765	1.274–2.445	0.001
rGFR（ml/min/1.73 m^2^)						
< 2	Ref.	–	–	–	–	–
2–5	1.427	0.197-10.36	0.73	–	–	–
≥5	2.155	0.301–15.425	0.44	–	–	–
peritonitis (times per month)	3.978	1.503–10.526	0.005	474.1	39.03–5759	<0.001
Albumin (per g/dL increment)	0.973	0.95–0.996	0.02	0.958	0.930–0.987	0.004
Kt/V (per 1 increment)	0.937	0.756–1.161	0.55	0.902	0.700–1.163	0.428
P (mmol/L)^a^						
<1.13	1.120	0.650–1.932	0.683	1.612	0.901–2.884	0.107
1.13–1.45	Ref.	–	–	Ref.	–	–
≥1.45	0.950	0.685–1.319	0.760	1.579	1.102–2.262	0.013

Note: adjusted for age, diabetes mellitus, eGFR, peritonitis, albumin, KT/V.

PD: peritoneal dialysis; HR: hazard ratio; CI: confidence interval; DM: diabetes mellitus; rGFR: estimated glomerular filtration rate; P: serum phosphorus; Kt/V: urea clearance index.

^a^Using the average value in the first PD year for P.

**Figure 2.**
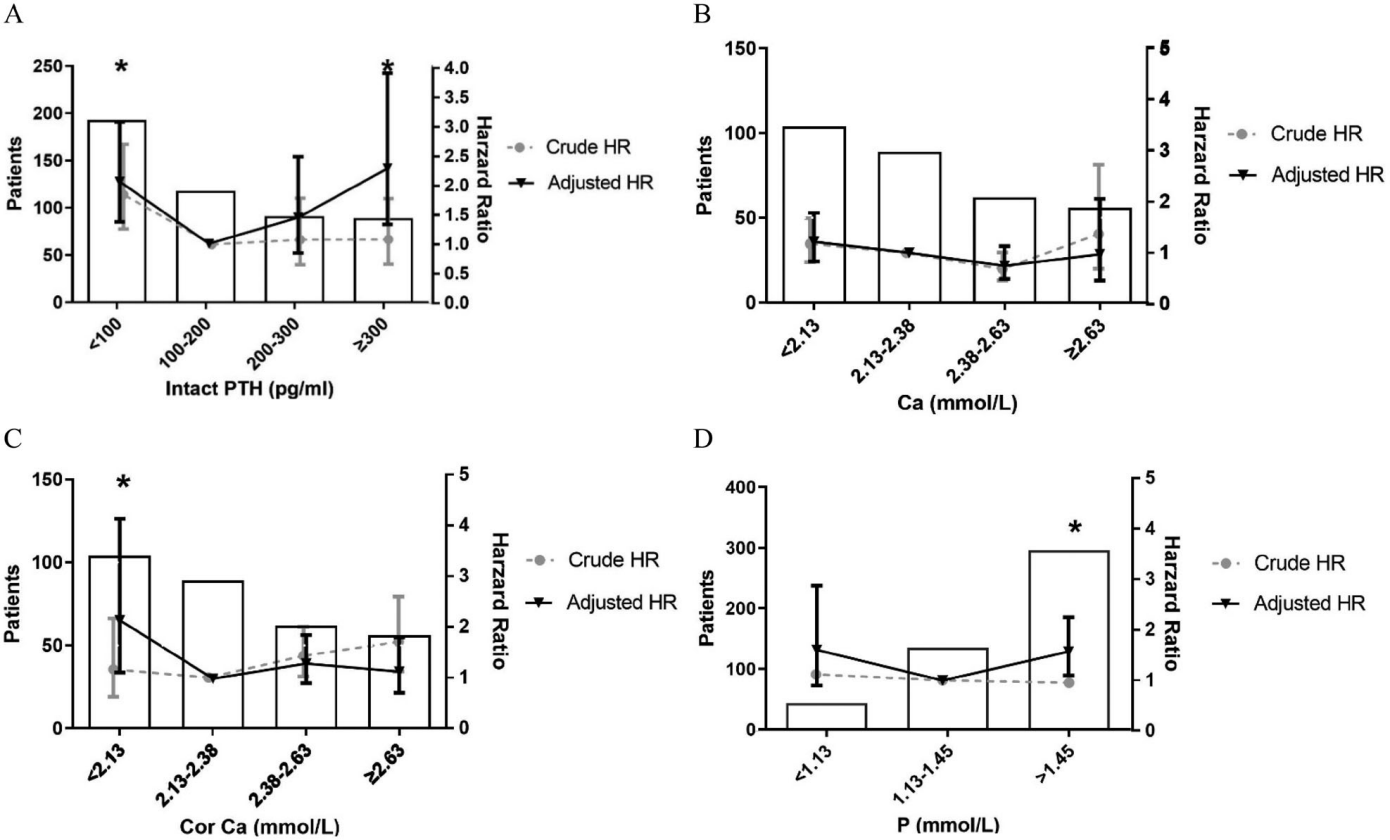
Cox Proportional Model Showing the Hazard Ratios of CKD-MBD Biomarkers to All-cause Mortality in Patients on Peritoneal Dialysis in PUMCH. Notes: Cox proportional regression model was adjusted for age, diabetes mellitus, rGFR, peritonitis, albumin and Kt/V. (A) Grouped by serum PTH levels, (B) Grouped by Ca levels, (C) Grouped by CorCa levels, (D) Grouped by P levels. CKD-MBD: chronic kidney disease-mineral bone disorder; eGFR: estimated glomerular filtration rate before PD start; PD: peritoneal dialysis; Kt/V: urea clearance index; Ca: serum total calcium; CorCa: corrected serum total calcium; P: serum phosphorus; PTH: parathyroid hormone. *Statistically significant difference compared with 100–200 pg/mL for PTH (A), 2.13–2.38 mmol/L for Ca (B), 2.13–2.38 mmol/L for CorCa (C), 1.13–1.45 mmol/L for P (D).

We observed a U-shaped association between PTH concentration and mortality in PD patients, with PTH concentrations of less than 100 pg/mL and 300 pg/mL or more being associated with increased all-cause mortality (reference PTH: 100 to <200 pg/mL). Hazard ratios and 95% CIs were 1.97 (1.32 to 2.94), 1.43 (0.83 to 2.44), and 2.24 (1.32 to 3.81) for PTH concentrations of <100 pg/mL, 200 to 300 pg/mL, and ≥300 pg/mL respectively (Table 2; Figure 2(A)). However, this pattern was not observed in serum calcium and phosphorus. In contrast, serum phosphorus at the levels of greater than 1.45 mmol/L were associated with higher all-cause mortality compared to that of reference range (1.13 to 1.45 mmol/L), and no significant difference was found for <1.13 mmol/L (Table 4; Figure 2(D)). Additionally, after adjustment for age, diabetes mellitus, rGFR, peritonitis, albumin and Kt/V, serum calcium was not a predictor for all-cause mortality (Figure 2(B)). However, CorCa <2.13 mmol/L exhibited higher mortality compared to reference range (2.13 to 2.38 mmol/L) (Table 3; Figure 2(C)).

In subgroup analysis, U-shaped association between PTH level and all-cause mortality was found in both diabetes mellitus (DM) group and non-Diabetes Mellitus (NDM) group (Supplementary Table 2). Furthermore, when we integrated CorCa, phosphorus, and PTH into the above multivariate Cox regression model simultaneously, phosphorus and PTH were still significant predictors for all-cause mortality (Supplementary Table 3). Phosphorus greater than 1.45 mmol/L showed increased mortality than the reference range (1.13 to 1.45 mmol/L).

### CKD-MBD Markers and Prognosis

**Figure 3.**
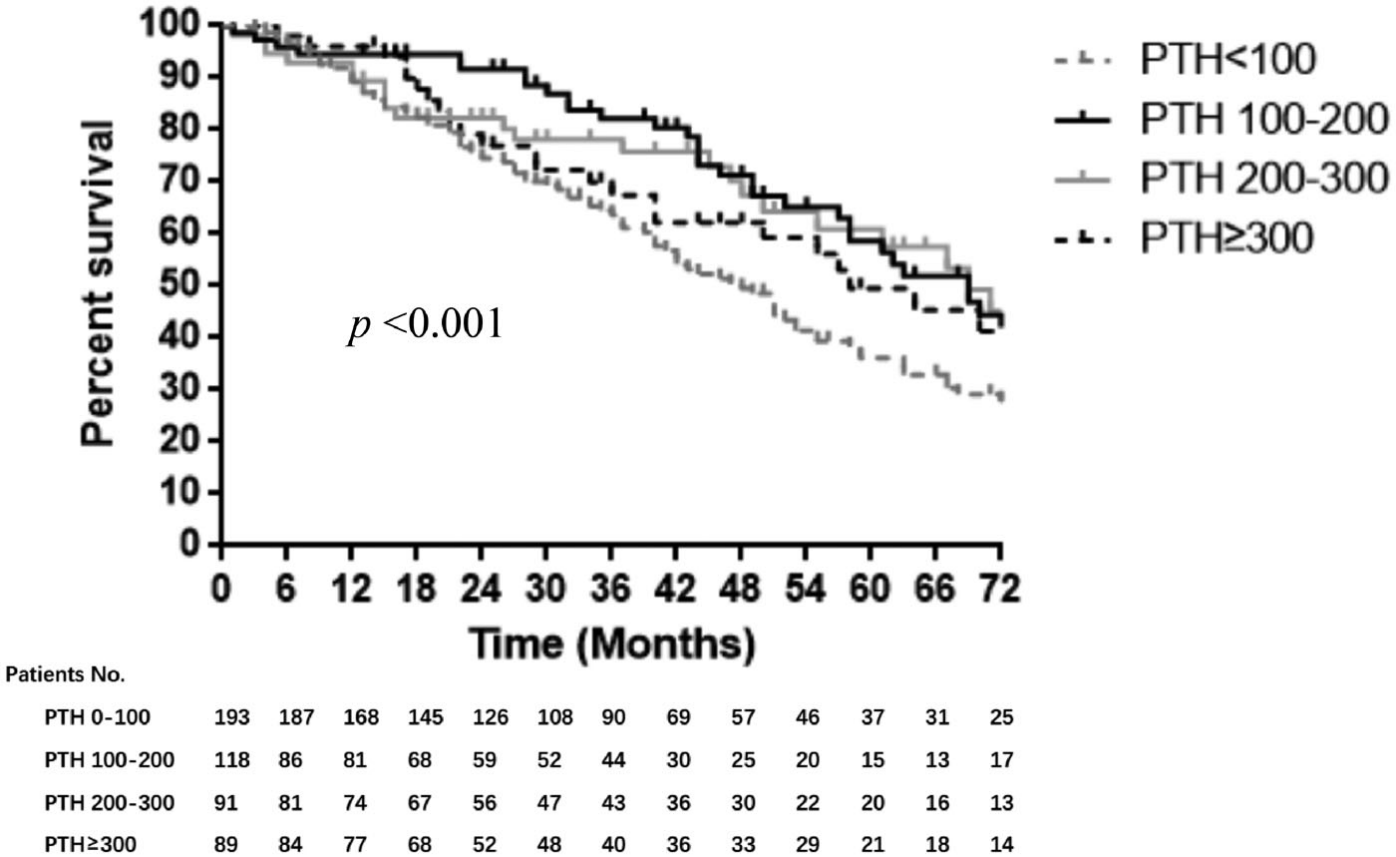
Kaplan-Meier Curves Showing the Survival of Peritoneal Dialysis Patients in PUMCH categorized by PTH (the average value in the first PD year). Notes: Significance was tested by log-rank test. PUMCH: Peking Union Medical College Hospital; PD: peritoneal dialysis; PTH: parathyroid hormone. Multiple comparison was performed by Sidak correction. *Statistically significant difference compared with 100–200 pg/mL.

In the Kaplan–Meier analyses, the 5-year survival rates were 42.4%, 66.4%, 69.3% and 63.5% for PTH level of <100, 100–200, 200–300 and ≥ 300 pg/mL respectively. There was an obvious difference in survival among these groups verified by Log-rank test (*p* < 0.001) (Figure 3). PTH level <100 pg/mL showed a significant lower survival probability compared to that of 100–200 pg/mL (*p* = 0.001). No significant difference in 5-year survival rates was observed among groups with different CorCa or phosphorus levels.

## Discussion

In this one of the largest PD cohort focusing on MBD to date, we first observed a *U*-shaped association between the average PTH levels of the first PD year and all-cause mortality. A significant association between lower serum CorCa level (<2.13 mmol/L) and higher serum phosphorus (≥1.45 mmol/L) were also found to predict all-cause mortality.

We first observed a U-shaped association of PTH with a concentration of 100–300 pg/mL exhibiting the lowest all-cause mortality in Chinese PD patients. Similar result was found in another study in Chinese incident HD patients, which concluded that the relationship between PTH and mortality also appears to be a U-shaped curve, and the optimal PTH level which confers the lowest risk of all-cause and cardiovascular mortality ranges from 150 pg/mL to 450 pg/mL [14]. Nonetheless, the treatment target of CKD-MBD in dialysis patients has been heatedly debated for decades. 2017 Kidney Disease: Improving Global Outcomes (KDIGO) guideline [6] recommends maintaining intact PTH levels in the range of approximately 2 to 9 times the upper normal limit for the assay in patients with CKD G5D. This 2C-level recommendation was based on evidence from HD patients with only a few Asian patients from Japan being included. Nevertheless, 2013 Japanese Society for Dialysis Therapy/Japanese Society of Nephrology (JSDT/JSN) suggests the target range of intact PTH is set between 60 and 240 pg/mL for an evidence level of 2D, which was also based on HD patients. In fact, no consensus has been reached for the relationship between PTH and mortality and the optimal PTH level in dialysis patients, especially in PD patients. Some HD studies showed that only higher PTH is associated with increased risk of mortality [2,15,16], while others conversely suggested that only lower PTH is detrimental [17,18]. Moreover, a few studies have shown a *U*-shaped relationship [19–22] or no significant association [8] between PTH and all-cause mortality. Inconsistency also exists in the already limited literature in PD patients. Race, nation and different approaches to handling iPTH (intact parathyroid hormone) value in the Cox regression analysis may be the major contributor. Rhee *et al.* studied data from 9,244 patients and demonstrated that PTH between 200–700 pg/mL exhibited the lowest mortality [10]. Rhee used the average PTH of the whole dialysis vintage (up to 20 years), where only 33% of their patients (68.9% in our study) was followed for more than 2 years. Considering individual PTH may dramatically change over the course due to MBD progression, using average PTH levels of the whole dialysis vintage in a retrospective cohort with patients of highly-varied length of follow-up might contribute to the bias. Recently, a study from Taiwan patients using the average PTH value of the first year showed that PTH level was not associated with mortality [11], which suggests high heterogeneity even with the same ethnic background. Younger age, fewer male and diabetes mellitus patients, and less mortality compared to our patients might contribute to such discrepancy as the former three factors are also known PTH predictors, illustrating potential effect of other clinical and laboratory indicators. In our study, patients with PTH levels of <100 pg/mL or ≥300 pg/mL had higher mortality than those with iPTH levels of 100–200 pg/mL at any given level of serum calcium or phosphate, which was more in line with 2003 KDOQI guideline [23]‘s recommendation on PTH level of 150–300 pg/mL.

Interestingly, the same U-shaped association between PTH level and all-cause mortality was found in both DM group and NDM group. DM patients on maintenance dialysis are often characterized by a relative PTH deficiency and adynamic bone [24,25]. Muras K *et al.* compared the effect of a 6-day high-phosphate diet on PTH in DM and non-DM CKD patients and observed that the PTH concentration was significantly higher on day 7 vs baseline in the DM group, but no increase in the non-DM group [26]. Although the mechanism is not clear, it might be due to the differences among the interaction of bone cells, and lead to lower bone turnover in the DM group [24]. Therefore, the same conclusion in both DM and NDM patients indicated the universality of the optimal level of PTH in clinical practice.

We also found a significant association between lower serum CorCa level (<2.13 mmol/L) or higher serum phosphorus (≥1.45 mmol/L) and increased mortality. There was always controversy on how serum calcium and phosphorus concentrations were associated with all-cause mortality. Data from most observational HD studies had shown that both serum phosphorus and calcium levels had U-shaped relationships with mortality [20,27,28]. Moreover, some studies indicated that hypercalcemia and hypophosphatemia or hyperphosphatemia predicted mortality [29,30]. The 2003 KDOQI CKD-MBD guideline [23] had proposed calcium level target to be 2.1–2.38 mmol/L, and phosphate at 1.13–1.78 mmol/L in dialysis patients. But this recommendation was not supported by a later retrospective cohort study from the United Kingdom [8], which included 4,947 hemodialysis and 2,129 peritoneal dialysis patients. In another prospective cohort study involving 586 PD patients, Noordij *et al.* demonstrated that only hyperphosphatemia more than 1.78 mmol/L, but not abnormal calcium levels or phosphate <1.13 mmol/L, were associated with increased mortality [31]. Similarly, we also found out that hyperphosphatemia was associated with increased mortality, but at a different cutoff level of 1.45 mmol/L (the upper limit of normal range in Chinese people). This is consistent with 2017 KDIGO guideline, which suggested lowering elevated phosphate levels toward the normal range beneficial, but not necessarily to further lower phosphate within the normal range [7]. Contrary to Liu et al’s finding in Taiwan that serum phosphorus levels <1.13 mmol/L was associated with increased mortality both the time-averaged value and the average value of the first year [11], both our data and Noordij *et al.* [30] failed to find the relationship between hypophosphatemia and mortality, possibly due to fewer patients (45/491 in our study and 53/586 in Noordij’s study) with phosphate <1.13 mmol/L. Other explanation might be diurnal variation in serum phosphate concentrations and variability among laboratories.

In the case of hypocalciumea, several speculations arise to explain its significant association with mortality in our study. Apart from arrhythmia, tumble, and fracture caused by hypocalcemia, patients with low serum calcium level usually had hyperphosphatemia which has been proved to be correlated with increased mortality. Doctors would prescribe more calcium supplements, vitamin D or its analog which in turn results in calcium overloading and leads to ectopic calcification. Therefore, hypocalcemia could also be an important risk factor for increased mortality. However, abnormal serum CorCa had no significant association with mortality in PD patients in Noordij *et al.* [31] and Stevens *et al.* [32]’s result. Liu’s study demonstrated serum CorCa level of ≥2.38 mmol/L was associated with increased mortality using the time-averaged value, while the average value of first-year serum CorCa level of 2.38–2.63 mmol/L was associated with increased mortality in Taiwan PD patients [11]. The differences among the optimal ranges of serum CorCa level found in different studies might be explained by different ethnics, regions, sunshine duration, and the heterogeneity of the different cutoff reference values used in each study. In the current literature, almost all studies used CorCa corrected by the serum albumin to predict the mortality instead of total serum Ca thus limited literature was available to compare with our results in total calcium.

There were several limitations in the present study. First, our study was a single-center, retrospective cohort study with a limited sample size which might affect the representativeness of Chinese PD patients. Second, other CKD-MBD markers associated with PTH, like serum 25-hydroxy vitamin D, alkaline phosphatase (ALP) and fibroblast growth factor-23 (FGF-23), were not available in this study. Third, information on medication and dialysate calcium concentration were absent from our study, which might contribute to residual confounding. In addition, a small portion (12%) of the patients had follow-ups <1 year, which might be short for long-term survival assessment. However, the main strengths of this study include the use of a database with PD patients in the same single center, making laboratory data strictly comparable, and the use of time-average CKD-MBD markers of the first PD year in the statistical analyses.

## Conclusions

Our study first demonstrated that best outcomes in Chinese PD patients have been observed in the PTH ranges of 100-300pg/ml, based on a U-shaped association between the average PTH levels of the first PD year and all-cause mortality. Both elevated serum phosphorus and decreased calcium were associated with increased all-cause mortality in PD patients. Further studies are needed to provide evidence for establishing a guideline for Chinese PD population.
